# *catena*-Poly[[[diaqua­bis(1*H*-indole-3-acetato-κ*O*)cobalt(II)]-μ-4,4′-bipyridine-κ^2^ *N*:*N*′] tetra­hydrate]

**DOI:** 10.1107/S1600536808010842

**Published:** 2008-04-23

**Authors:** Ji-Wei Liu, Seik Weng Ng

**Affiliations:** a College of Chemistry and Chemical Technology, Daqing Petroleum Institute, Daqing 163318, People’s Republic of China; b Department of Chemistry, University of Malaya, 50603 Kuala Lumpur, Malaysia

## Abstract

The 4,4′-bipyridine spacer in the title compound, [Co(C_10_H_8_NO_2_)_2_(C_10_H_8_N_2_)(H_2_O)_2_]·4H_2_O, links the diaqua­cobalt(II) dicarboxyl­ate units into a linear chain; the metal atom lies on a center of inversion in an octa­hedral environment. The coordinated and uncoordinated water mol­ecules inter­act through O—H⋯O hydrogen bonds to form a three-dimensional network.

## Related literature

For examples of diaqua transition-metal dicarboxyl­ates that are linked by 4,4′-bipyridine, see: Deng *et al.* (2005 ▶); Gao *et al.* (2006 ▶); He *et al.* (2003 ▶); Hou *et al.* (2007 ▶); Li *et al.* (2006 ▶); Pedireddi & Varughese (2004 ▶); Yan *et al.* (2005 ▶); Zhang *et al.* (1999 ▶); Zheng, Su & Feng (2006 ▶); Zheng, Tong & Chen (2006 ▶).
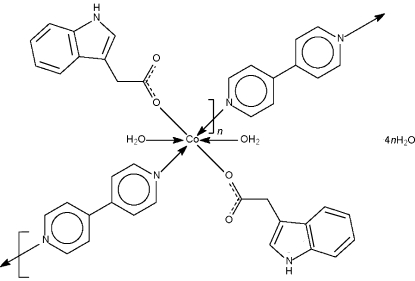


## Experimental

### 

#### Crystal data

[Co(C_10_H_8_NO_2_)_2_(C_10_H_8_N_2_)(H_2_O)_2_]·4H_2_O
*M* *_r_* = 671.56
Monoclinic, 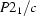

*a* = 11.5011 (3) Å
*b* = 16.0953 (4) Å
*c* = 8.8302 (2) Å
β = 111.784 (1)°
*V* = 1517.86 (6) Å^3^
*Z* = 2
Mo *K*α radiation
μ = 0.63 mm^−1^
*T* = 295 (2) K
0.35 × 0.20 × 0.08 mm

#### Data collection

Bruker APEXII diffractometer
Absorption correction: multi-scan (*SADABS*; Sheldrick, 1996 ▶) *T*_min_ = 0.810, *T*_max_ = 0.951
11131 measured reflections
2584 independent reflections
1771 reflections with *I* > 2˘*I*)
*R*_int_ = 0.037

#### Refinement

*R*[*F* ^2^ > 2σ(*F* ^2^)] = 0.040
*wR*(*F* ^2^) = 0.102
*S* = 1.03
2584 reflections
226 parameters
10 restraints
H atoms treated by a mixture of independent and constrained refinement
Δρ_max_ = 0.33 e Å^−3^
Δρ_min_ = −0.53 e Å^−3^

### 

Data collection: *APEX2* (Bruker, 2006 ▶); cell refinement: *SAINT* (Bruker, 2006 ▶); data reduction: *SAINT*; program(s) used to solve structure: *SHELXS97* (Sheldrick, 2008 ▶); program(s) used to refine structure: *SHELXL97* (Sheldrick, 2008 ▶); molecular graphics: *X-SEED* (Barbour, 2001 ▶); software used to prepare material for publication: *SHELXL97*.

## Supplementary Material

Crystal structure: contains datablocks global, I. DOI: 10.1107/S1600536808010842/sg2234sup1.cif

Structure factors: contains datablocks I. DOI: 10.1107/S1600536808010842/sg2234Isup2.hkl

Additional supplementary materials:  crystallographic information; 3D view; checkCIF report

## Figures and Tables

**Table:** 

Co1—O1	2.182 (2)
Co1—O1*W*	2.042 (2)
Co1—N2	2.184 (2)

**Table:** 

O1—Co1—O1*W*	89.74 (7)
O1—Co1—O1*W*^i^	90.26 (7)
O1—Co1—N2	91.58 (7)
O1—Co1—N2^i^	88.42 (7)
O1*W*—Co1—N2	91.63 (8)
O1*W*—Co1—N2^i^	88.37 (8)

Symmetry code: (i) 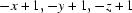.

**Table 2:** Hydrogen-bond geometry (Å, °)

*D*—H⋯*A*	*D*—H	H⋯*A*	*D*⋯*A*	*D*—H⋯*A*
O1*W*—H1*W*1⋯O2	0.85 (3)	1.76 (3)	2.585 (2)	165 (3)
O1*W*—H1*W*2⋯O2*W*	0.85 (3)	1.89 (3)	2.724 (3)	171 (3)
O2*W*—H2*W*1⋯O3*W*^ii^	0.85 (3)	1.89 (3)	2.735 (3)	173 (3)
O2*W*—H2*W*2⋯O2^iii^	0.84 (3)	1.94 (3)	2.752 (3)	162 (3)
O3*W*—H3*W*1⋯O1	0.85 (3)	2.00 (3)	2.854 (3)	179 (3)
O3*W*—H3*W*2⋯O2*W*^i^	0.85 (3)	2.03 (3)	2.872 (3)	168 (3)

Symmetry codes: (i) 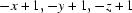; (ii) 
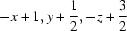
; (iii) 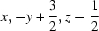.

**Fig. 1.**
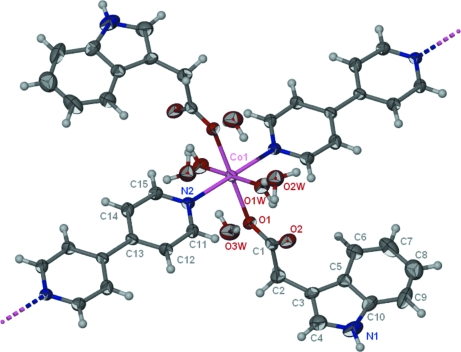
Thermal displacement ellipsoid plot (Barbour, 2001) illustrating the coordination geometry of cobalt in Co(C10H8N2)(C10H8NO2)2(H2O)2.4H2O. Displacement ellipsoids are drawn at the 50% probability level and H atoms as spheres of arbitrary radii.

## supplementary crystallographic information

### Comment

There are many crystallographic examples of transition metal di(carboxylates) that are linked by the 4,4'-bipyridine spacer ligand. Among these are examples that have coordinated water; the water entities engage in hydrogen bonding that consolidates the structure.

The title diaquadi(indole-3-carboxylato)cobalt–4,4'-bipyridine tetrahydrate (Scheme I) exists as a chain. The metal atom is coordinated by two unidentate carboxylate groups, two water molecules and two 4,4'-bipyridine ligands in an octahedral geometry (Fig. 1). The spacer ligand links the mononuclear units into a linear chain. The chains are further linked by hydrogen bonds into a three-dimensional network (Table 2).

### Experimental

Cobalt(II) sulfate septahydrate (0.28 g, 1 mmol) and 4,4'-bipyridine (0.16 g, 1 mmol) were added to a hot aqueous solution of indole-3-acetic acid (0.12 g, 1 mmol); the pH was adjusted to 6 with 0.1*M* sodium hydroxide. The solution was allowed to evaporate at room temperature. Single crystals are separated from the filtered solution after several days.

### Refinement

Hydrogen atoms were treated as riding, with C–H = 0.93 to 0.97 Å and were included in the refinement with *U*(H) set to 1.2 times *U*_eq_(C). The water and amino H-atoms were located in a difference Fourier map, and were refined with distance restraints of O–H = N–H = 0.85±0.01 Å and H···H 1.39±0.01 Å. Their temperature factors were tied to those of the parent atoms by a factor of 1.5

### Crystal data

**Table:**

[Co(C_10_H_8_NO_2_)_2_(C_10_H_8_N_2_)(H_2_O)_2_]·4H_2_O	*F*_000_ = 702
*M**_r_* = 671.56	*D*_x_ = 1.469 Mg m^−^^3^
Monoclinic, *P*2_1_/*c*	Mo *K*α radiation λ = 0.71073 Å
Hall symbol: -P 2ybc	Cell parameters from 2534 reflections
*a* = 11.5011 (3) Å	θ = 2.3–21.7º
*b* = 16.0953 (4) Å	µ = 0.63 mm^−^^1^
*c* = 8.8302 (2) Å	*T* = 295 (2) K
β = 111.784 (1)º	Block, pink
*V* = 1517.86 (6) Å^3^	0.35 × 0.20 × 0.08 mm
*Z* = 2	

### Data collection

**Table:**

Bruker APEXII diffractometer	2584 independent reflections
Radiation source: fine-focus sealed tube	1771 reflections with *I* > 2˘*I*)
Monochromator: graphite	*R*_int_ = 0.037
*T* = 295(2) K	θ_max_ = 25.0º
φ and ω scans	θ_min_ = 1.9º
Absorption correction: Multi-scan(SADABS; Sheldrick, 1996)	*h* = −13→13
*T*_min_ = 0.810, *T*_max_ = 0.951	*k* = −17→19
11131 measured reflections	*l* = −10→9

### Refinement

**Table:**

Refinement on *F*^2^	Secondary atom site location: difference Fourier map
Least-squares matrix: full	Hydrogen site location: inferred from neighbouring sites
*R*[*F*^2^ > 2σ(*F*^2^)] = 0.040	H atoms treated by a mixture of independent and constrained refinement
*wR*(*F*^2^) = 0.102	*w* = 1/[σ^2^(*F*_o_^2^) + (0.0506*P*)^2^ + 0.1356*P*] where *P* = (*F*_o_^2^ + 2*F*_c_^2^)/3
*S* = 1.03	(Δ/σ)_max_ = 0.001
2584 reflections	Δρ_max_ = 0.33 e Å^−^^3^
226 parameters	Δρ_min_ = −0.53 e Å^−^^3^
10 restraints	Extinction correction: none
Primary atom site location: structure-invariant direct methods	

### Fractional atomic coordinates and isotropic or equivalent isotropic displacement parameters (Å^2^)

**Table:**

	*x*	*y*	*z*	*U*_iso_*/*U*_eq_	
Co1	0.5000	0.5000	0.5000	0.02917 (19)	
O1	0.57914 (16)	0.50222 (11)	0.7661 (2)	0.0353 (5)	
O2	0.60753 (17)	0.63848 (12)	0.8108 (2)	0.0454 (5)	
O1W	0.50557 (18)	0.62679 (12)	0.4969 (2)	0.0423 (5)	
H1W1	0.539 (2)	0.6396 (16)	0.5967 (13)	0.063*	
H1W2	0.472 (2)	0.6689 (12)	0.441 (3)	0.063*	
O2W	0.4133 (2)	0.77242 (13)	0.3416 (3)	0.0535 (6)	
H2W1	0.387 (2)	0.7955 (17)	0.409 (3)	0.080*	
H2W2	0.4825 (18)	0.7928 (19)	0.348 (4)	0.080*	
O3W	0.6525 (2)	0.34501 (14)	0.9218 (3)	0.0617 (6)	
H3W1	0.631 (3)	0.3921 (10)	0.877 (3)	0.093*	
H3W2	0.644 (3)	0.3081 (13)	0.850 (3)	0.093*	
N1	0.8052 (3)	0.72774 (16)	1.3063 (3)	0.0603 (8)	
H1N	0.806 (3)	0.7654 (15)	1.373 (3)	0.090*	
N2	0.30936 (19)	0.50106 (13)	0.4973 (3)	0.0335 (6)	
C1	0.6121 (2)	0.56454 (19)	0.8587 (3)	0.0339 (7)	
C2	0.6562 (3)	0.55025 (19)	1.0415 (3)	0.0443 (8)	
H2A	0.5834	0.5440	1.0709	0.053*	
H2B	0.7028	0.4985	1.0676	0.053*	
C3	0.7364 (3)	0.61775 (17)	1.1427 (3)	0.0374 (7)	
C4	0.7058 (3)	0.6757 (2)	1.2330 (4)	0.0530 (9)	
H4	0.6285	0.6796	1.2436	0.064*	
C5	0.8624 (3)	0.63474 (17)	1.1623 (3)	0.0369 (7)	
C6	0.9462 (3)	0.5963 (2)	1.1030 (4)	0.0522 (8)	
H6	0.9216	0.5504	1.0344	0.063*	
C7	1.0646 (3)	0.6275 (3)	1.1475 (5)	0.0712 (11)	
H7	1.1208	0.6022	1.1088	0.085*	
C8	1.1028 (3)	0.6955 (3)	1.2485 (5)	0.0776 (12)	
H8	1.1842	0.7149	1.2761	0.093*	
C9	1.0246 (3)	0.7352 (2)	1.3093 (4)	0.0656 (10)	
H9	1.0510	0.7812	1.3771	0.079*	
C10	0.9034 (3)	0.70374 (18)	1.2654 (3)	0.0460 (8)	
C11	0.2700 (2)	0.55901 (17)	0.5739 (4)	0.0393 (7)	
H11	0.3255	0.6011	0.6273	0.047*	
C12	0.1515 (2)	0.56060 (17)	0.5789 (3)	0.0401 (7)	
H12	0.1300	0.6025	0.6362	0.048*	
C13	0.0644 (2)	0.50028 (16)	0.4993 (3)	0.0309 (6)	
C14	0.1058 (2)	0.44017 (18)	0.4190 (4)	0.0439 (8)	
H14	0.0521	0.3977	0.3633	0.053*	
C15	0.2258 (3)	0.44290 (18)	0.4212 (4)	0.0439 (8)	
H15	0.2503	0.4014	0.3659	0.053*	

### Atomic displacement parameters (Å^2^)

**Table:**

	*U*^11^	*U*^22^	*U*^33^	*U*^12^	*U*^13^	*U*^23^
Co1	0.0262 (3)	0.0343 (3)	0.0289 (3)	0.0001 (2)	0.0124 (2)	0.0008 (3)
O1	0.0385 (11)	0.0364 (11)	0.0311 (11)	−0.0045 (9)	0.0129 (9)	−0.0043 (10)
O2	0.0620 (14)	0.0377 (13)	0.0338 (12)	−0.0019 (11)	0.0146 (10)	0.0007 (11)
O1W	0.0498 (12)	0.0364 (12)	0.0347 (11)	0.0023 (10)	0.0088 (10)	0.0033 (10)
O2W	0.0640 (15)	0.0449 (14)	0.0537 (15)	−0.0024 (12)	0.0243 (12)	−0.0021 (11)
O3W	0.0847 (17)	0.0520 (14)	0.0557 (15)	0.0017 (15)	0.0343 (14)	0.0021 (12)
N1	0.081 (2)	0.0483 (19)	0.0477 (19)	0.0026 (17)	0.0200 (17)	−0.0148 (15)
N2	0.0311 (12)	0.0415 (14)	0.0303 (13)	−0.0024 (12)	0.0142 (10)	−0.0054 (12)
C1	0.0275 (14)	0.045 (2)	0.0317 (17)	−0.0034 (14)	0.0144 (12)	−0.0003 (17)
C2	0.0499 (18)	0.053 (2)	0.0284 (17)	−0.0093 (16)	0.0130 (14)	−0.0004 (16)
C3	0.0463 (17)	0.0416 (18)	0.0240 (16)	−0.0032 (15)	0.0125 (13)	−0.0028 (14)
C4	0.051 (2)	0.063 (2)	0.046 (2)	0.0047 (18)	0.0186 (16)	−0.0020 (18)
C5	0.0443 (17)	0.0373 (18)	0.0297 (16)	−0.0010 (15)	0.0145 (13)	0.0031 (15)
C6	0.056 (2)	0.054 (2)	0.052 (2)	−0.0043 (17)	0.0275 (17)	−0.0029 (17)
C7	0.059 (2)	0.087 (3)	0.078 (3)	−0.001 (2)	0.037 (2)	0.010 (2)
C8	0.053 (2)	0.102 (3)	0.071 (3)	−0.022 (2)	0.015 (2)	0.019 (3)
C9	0.073 (3)	0.058 (2)	0.049 (2)	−0.026 (2)	0.0027 (19)	0.0010 (19)
C10	0.059 (2)	0.0398 (19)	0.0331 (19)	−0.0060 (17)	0.0094 (16)	−0.0011 (16)
C11	0.0318 (16)	0.0437 (18)	0.0445 (19)	−0.0071 (14)	0.0166 (13)	−0.0130 (16)
C12	0.0348 (16)	0.0449 (19)	0.0467 (19)	−0.0023 (14)	0.0221 (14)	−0.0130 (15)
C13	0.0266 (14)	0.0405 (17)	0.0276 (15)	0.0003 (14)	0.0125 (11)	−0.0003 (14)
C14	0.0317 (16)	0.049 (2)	0.054 (2)	−0.0066 (14)	0.0191 (14)	−0.0151 (16)
C15	0.0361 (17)	0.052 (2)	0.049 (2)	−0.0035 (16)	0.0225 (15)	−0.0193 (17)

### Geometric parameters (Å, °)

**Table:**

Co1—O1	2.182 (2)	C3—C4	1.355 (4)
Co1—O1^i^	2.182 (2)	C3—C5	1.421 (3)
Co1—O1W^i^	2.042 (2)	C4—H4	0.9300
Co1—O1W	2.042 (2)	C5—C6	1.400 (4)
Co1—N2	2.184 (2)	C5—C10	1.402 (4)
Co1—N2^i^	2.184 (2)	C6—C7	1.365 (4)
O1—C1	1.260 (3)	C6—H6	0.9300
O2—C1	1.258 (3)	C7—C8	1.376 (5)
O1W—H1W1	0.85 (3)	C7—H7	0.9300
O1W—H1W2	0.85 (3)	C8—C9	1.365 (5)
O2W—H2W1	0.85 (3)	C8—H8	0.9300
O2W—H2W2	0.84 (3)	C9—C10	1.396 (4)
O3W—H3W1	0.85 (3)	C9—H9	0.9300
O3W—H3W2	0.85 (3)	C11—C12	1.380 (3)
N1—C10	1.364 (4)	C11—H11	0.9300
N1—C4	1.371 (4)	C12—C13	1.385 (3)
N1—H1N	0.84 (1)	C12—H12	0.9300
N2—C11	1.327 (3)	C13—C14	1.384 (3)
N2—C15	1.331 (3)	C13—C13^ii^	1.487 (5)
C1—C2	1.519 (4)	C14—C15	1.374 (3)
C2—C3	1.489 (4)	C14—H14	0.9300
C2—H2A	0.9700	C15—H15	0.9300
C2—H2B	0.9700		
			
O1—Co1—O1^i^	180.0	N1—C4—H4	124.9
O1—Co1—O1W	89.74 (7)	C6—C5—C10	118.7 (3)
O1—Co1—O1W^i^	90.26 (7)	C6—C5—C3	133.0 (3)
O1—Co1—N2	91.58 (7)	C10—C5—C3	108.3 (2)
O1—Co1—N2^i^	88.42 (7)	C7—C6—C5	118.8 (3)
O1W—Co1—O1W^i^	180.0	C7—C6—H6	120.6
O1W—Co1—N2	91.63 (8)	C5—C6—H6	120.6
O1W—Co1—N2^i^	88.37 (8)	C6—C7—C8	121.6 (3)
N2—Co1—N2^i^	180.0	C6—C7—H7	119.2
C1—O1—Co1	128.05 (17)	C8—C7—H7	119.2
Co1—O1W—H1W1	104 (2)	C9—C8—C7	121.9 (3)
Co1—O1W—H1W2	143 (2)	C9—C8—H8	119.1
H1W1—O1W—H1W2	111 (2)	C7—C8—H8	119.1
H2W1—O2W—H2W2	111 (2)	C8—C9—C10	117.2 (3)
H3W1—O3W—H3W2	110 (2)	C8—C9—H9	121.4
C10—N1—C4	109.2 (3)	C10—C9—H9	121.4
C10—N1—H1N	127 (3)	N1—C10—C9	131.6 (3)
C4—N1—H1N	124 (3)	N1—C10—C5	106.5 (3)
C11—N2—C15	115.6 (2)	C9—C10—C5	121.9 (3)
C11—N2—Co1	122.10 (17)	N2—C11—C12	123.9 (2)
C15—N2—Co1	122.25 (17)	N2—C11—H11	118.1
O2—C1—O1	124.7 (3)	C12—C11—H11	118.1
O2—C1—C2	117.2 (3)	C11—C12—C13	120.5 (2)
O1—C1—C2	118.1 (2)	C11—C12—H12	119.7
C3—C2—C1	114.6 (2)	C13—C12—H12	119.7
C3—C2—H2A	108.6	C14—C13—C12	115.4 (2)
C1—C2—H2A	108.6	C14—C13—C13^ii^	122.2 (3)
C3—C2—H2B	108.6	C12—C13—C13^ii^	122.4 (3)
C1—C2—H2B	108.6	C15—C14—C13	120.3 (3)
H2A—C2—H2B	107.6	C15—C14—H14	119.8
C4—C3—C5	105.8 (3)	C13—C14—H14	119.8
C4—C3—C2	128.1 (3)	N2—C15—C14	124.2 (3)
C5—C3—C2	126.1 (2)	N2—C15—H15	117.9
C3—C4—N1	110.2 (3)	C14—C15—H15	117.9
C3—C4—H4	124.9		
			
O1W^i^—Co1—O1—C1	178.5 (2)	C2—C3—C5—C10	179.3 (3)
O1W—Co1—O1—C1	−1.5 (2)	C10—C5—C6—C7	−0.1 (4)
N2—Co1—O1—C1	−93.1 (2)	C3—C5—C6—C7	−178.7 (3)
N2^i^—Co1—O1—C1	86.9 (2)	C5—C6—C7—C8	−0.2 (5)
O1W^i^—Co1—N2—C11	144.9 (2)	C6—C7—C8—C9	0.1 (6)
O1W—Co1—N2—C11	−35.1 (2)	C7—C8—C9—C10	0.2 (5)
O1—Co1—N2—C11	54.6 (2)	C4—N1—C10—C9	−179.1 (3)
O1^i^—Co1—N2—C11	−125.4 (2)	C4—N1—C10—C5	0.4 (3)
O1W^i^—Co1—N2—C15	−34.0 (2)	C8—C9—C10—N1	178.9 (3)
O1W—Co1—N2—C15	146.0 (2)	C8—C9—C10—C5	−0.5 (5)
O1—Co1—N2—C15	−124.2 (2)	C6—C5—C10—N1	−179.2 (3)
O1^i^—Co1—N2—C15	55.8 (2)	C3—C5—C10—N1	−0.2 (3)
Co1—O1—C1—O2	−2.0 (4)	C6—C5—C10—C9	0.4 (4)
Co1—O1—C1—C2	175.64 (15)	C3—C5—C10—C9	179.4 (3)
O2—C1—C2—C3	−24.1 (3)	C15—N2—C11—C12	0.7 (4)
O1—C1—C2—C3	158.1 (2)	Co1—N2—C11—C12	−178.1 (2)
C1—C2—C3—C4	106.8 (3)	N2—C11—C12—C13	−1.0 (4)
C1—C2—C3—C5	−72.4 (4)	C11—C12—C13—C14	0.6 (4)
C5—C3—C4—N1	0.4 (3)	C11—C12—C13—C13^ii^	−179.7 (3)
C2—C3—C4—N1	−179.0 (3)	C12—C13—C14—C15	−0.2 (4)
C10—N1—C4—C3	−0.5 (4)	C13^ii^—C13—C14—C15	−179.8 (3)
C4—C3—C5—C6	178.7 (3)	C11—N2—C15—C14	−0.2 (4)
C2—C3—C5—C6	−2.0 (5)	Co1—N2—C15—C14	178.7 (2)
C4—C3—C5—C10	−0.1 (3)	C13—C14—C15—N2	−0.1 (5)

Symmetry codes: (i) −*x*+1, −*y*+1, −*z*+1; (ii) −*x*, −*y*+1, −*z*+1.

### Hydrogen-bond geometry (Å, °)

**Table:**

*D*—H···*A*	*D*—H	H···*A*	*D*···*A*	*D*—H···*A*
O1W—H1W1···O2	0.85 (3)	1.76 (3)	2.585 (2)	165 (3)
O1W—H1W2···O2W	0.85 (3)	1.89 (3)	2.724 (3)	171 (3)
O2W—H2W1···O3W^iii^	0.85 (3)	1.89 (3)	2.735 (3)	173 (3)
O2W—H2W2···O2^iv^	0.84 (3)	1.94 (3)	2.752 (3)	162 (3)
O3W—H3W1···O1	0.85 (3)	2.00 (3)	2.854 (3)	179 (3)
O3W—H3W2···O2W^i^	0.85 (3)	2.03 (3)	2.872 (3)	168 (3)
N1—H1N···O2^v^	0.84 (1)	2.64 (3)	3.142 (3)	120 (3)

Symmetry codes: (iii) −*x*+1, *y*+1/2, −*z*+3/2; (iv) *x*, −*y*+3/2, *z*−1/2; (i) −*x*+1, −*y*+1, −*z*+1; (v) *x*, −*y*+3/2, *z*+1/2.
